# Quasi-Bound State in the Continuum-Driven Co-Frequency Polarization-Multiplexed Tunable Terahertz All Dielectric Metasurface

**DOI:** 10.3390/nano16191257

**Published:** 2026-10-05

**Authors:** Xilai Zhao, Jiahe Wang, Yifang Yuan, Jiangang Liang

**Affiliations:** 1 Air and Missile Defense College, Air Force Engineering University, Xi’an 710051, China; zxl511178738@126.com (X.Z.); wangjiahe2025@163.com (J.W.); 2 School of Electronics and Information Engineering, Anhui University, Hefei 230601, China

**Keywords:** quasi-bound states in the continuum, all-dielectric terahertz metasurface, active modulation, polarization multiplexing, Fano resonance, electromagnetically induced transparency

## Abstract

Quasi-bound states in the continuum (q-BICs) provide an effective approach for achieving strongly confined resonances in metasurfaces. However, the simultaneous realization of high-Q, polarization-multiplexed, and tunable resonances at a single frequency using a single resonant meta-atom remains challenging. Here, we numerically design and investigate a tunable all-dielectric terahertz (THz) metasurface consisting of a single asymmetric germanium (Ge) resonator by employing COMSOL Multiphysics. By introducing only one asymmetry parameter, the symmetry-protected BIC is transformed into a radiative q-BIC under two orthogonal linear polarizations. Through the coupling between the q-BIC and the intrinsic resonant mode, distinct interference responses are generated. Specifically, at the same frequency of 1.227 THz, a Fano resonance (Q = 606) is obtained under TE polarization, whereas an electromagnetically induced transparency (EIT)-like resonance (Q = 1105) appears under TM polarization. Owing to the tunable property of Ge, the two resonances can be actively modulated, achieving modulation depths of 96.7% and 99.1%, respectively. Furthermore, under TM polarization, the group delay can be continuously tuned from 0 to 259 ps by controlling the Ge conductivity. The proposed metasurface provides a compact platform for polarization-multiplexed and dynamically tunable THz photonic devices.

## 1. Introduction

THz electromagnetic waves have attracted considerable interest owing to their unique advantages, including nonionizing radiation, broad bandwidth, and high temporal and spatial resolutions [1,2,3]. As subwavelength platforms that exploit diverse resonances for efficient wave manipulation, metasurfaces hold great promise for the precise and customized control of THz radiation [4,5]. In particular, high-Q resonances are of fundamental importance for enhancing the spectral selectivity, field localization, and light–matter interaction in THz metasurfaces [6,7]. In this context, all-dielectric devices have received considerable attention because they can support various resonant responses with relatively low material losses. Among these, a bound state in the continuum (BIC) is a localized mode embedded in the radiation continuum while remaining decoupled from free-space radiation, resulting in an ideally infinite Q factor [8,9]. In practical structures, a symmetry-protected BIC can be converted into a radiative (quasi-bound state in the continuum) q-BIC by introducing structural asymmetry. The resulting state exhibits a finite radiative linewidth and a high yet experimentally accessible Q factor [8,9,10]. Therefore, the asymmetry parameter provides an effective means of controlling the radiative leakage and spectral linewidth of the resonance. To date, q-BIC-based THz metasurfaces have shown considerable potential for applications such as narrowband filters, high-precision imaging, and sensitive biosensing [11,12,13,14,15].

More interestingly, the interaction between a q-BIC and another mode supported by the same dielectric resonator can generate more diverse spectral responses. When two resonant channels interfere, an asymmetric Fano resonance or a narrow transmission window analogous to Electromagnetically induced transparency (EIT) can be produced [16,17,18]. Fano resonances are generally accompanied by steep spectral variations and strong amplitude contrast, whereas EIT-like resonances exhibit pronounced dispersion and enhanced group delay. These characteristics further extend the potential applications of q-BIC-based THz metasurfaces [19,20,21,22,23]. However, achieving such effects often relies on the interaction between multiple resonators, which increases the complexity of device design and fabrication. In addition, owing to the complex resonant behavior introduced by multiple asymmetry parameters, existing q-BIC metasurfaces are often polarization-sensitive. Furthermore, modulation of q-BICs generally relies on changes in structural parameters, limiting the dynamic tunability of the devices. Some previous studies have employed tunable materials to control q-BIC-based THz metasurfaces, including hybrid semiconductors [24], phase change materials [14,25], and two-dimensional materials [19,26,27]. However, introducing additional functional materials into the unit cell requires careful consideration of interfacial compatibility, lattice matching, and the suitability of the external stimulation method. Moreover, the carrier relaxation time of semiconductors such as silicon typically ranges in nanoseconds, whereas phase-change materials generally exhibit slower responses. Since THz waves operate on a picosecond timescale, the response speed of these materials may be insufficient for THz devices. Ge, whose conductivity can be tuned over a broad range under optical modulation and whose carrier relaxation time is on the order of a few picoseconds, is therefore a promising dielectric material platform for THz applications [5,28,29]. Moreover, Ge is relatively abundant and can be processed using well-established techniques, potentially reducing fabrication costs compared with two-dimensional materials such as graphene. As a result, engineering the coupling between q-BICs and different resonant modes within a single asymmetric Ge resonator may offer a compact route to reducing structural complexity while enabling polarization-multiplexed resonant responses and active conductivity-controlled tuning.

In this work, we propose and numerically investigate a tunable all-dielectric THz metasurface composed of Ge resonators supported by a silica (SiO_2_) substrate by employing COMSOL Multiphysics 6.0. A single in-plane symmetry-breaking parameter is introduced into each Ge resonator to transform symmetry-protected BICs into radiative quasi-BICs. By tailoring the resonant coupling under two orthogonal linear polarizations, the metasurface realizes a co-frequency polarization-multiplexed response at 1.227 THz: a Fano resonance is excited under TE polarization, whereas an EIT-like transparency response is obtained under TM polarization. Although the two channels share the same operating frequency, their distinct spectral line shapes and phase responses enable polarization-selective functional operation. Benefiting from the photoinduced conductivity variation of Ge, both resonances can be actively suppressed with modulation depths of 96.7% and 99.1% under TE and TM excitation, respectively. In addition, the TM-channel EIT-like response provides a continuously tunable group delay from 0 to 259 ps. The proposed single-resonator platform therefore offers a compact route toward co-frequency polarization-multiplexed, actively reconfigurable THz devices for narrowband modulation, slow-light control, and sensing.

## 2. Materials and Methods

**Figure 1:**
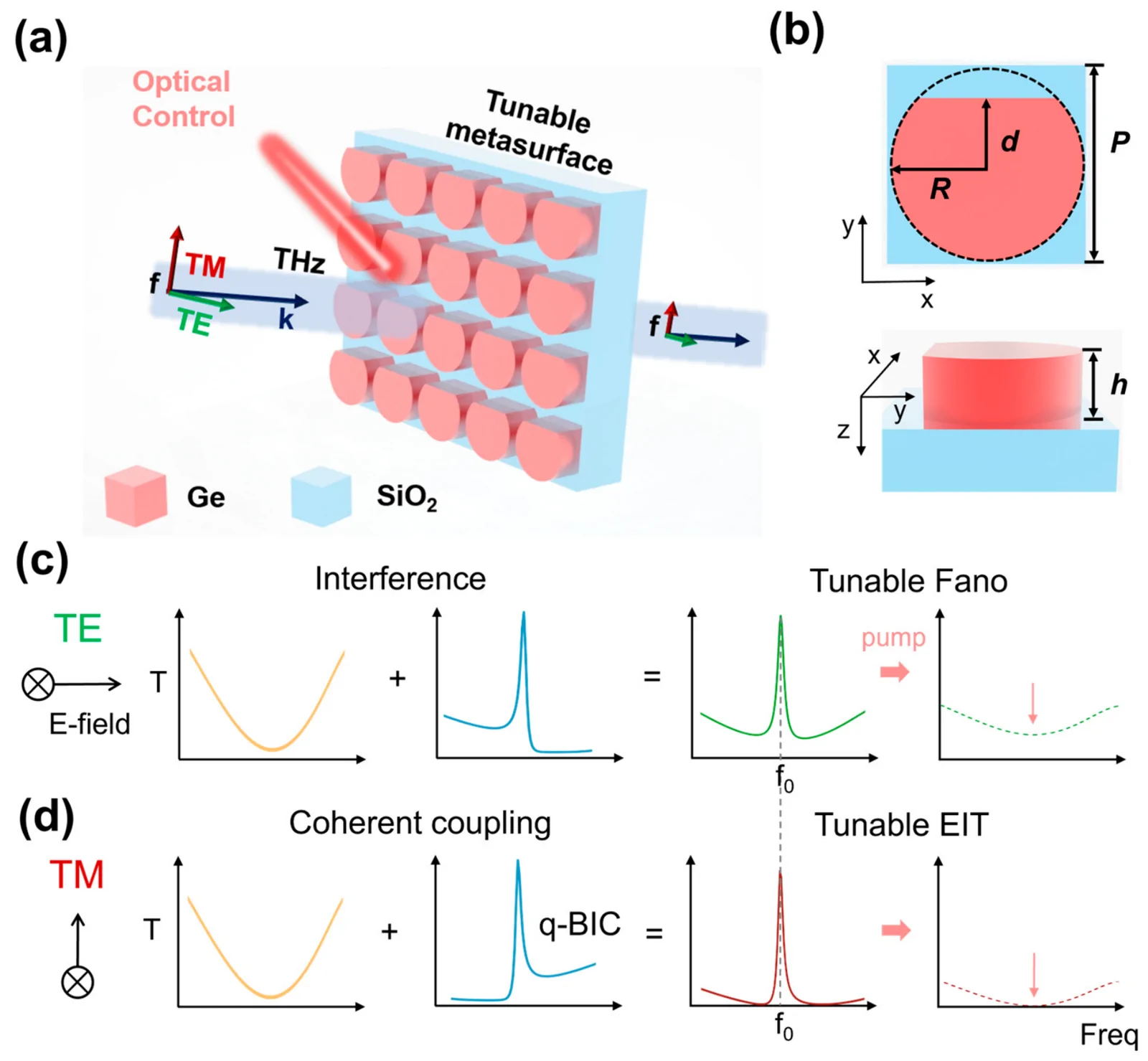
Construction and operating principle of the THz tunable metasurface. (**a**) Schematic and principle of the all-dielectric metasurface composed of an asymmetric Ge cylinder on a SiO_2_ substrate. (**b**) Unit cell structure with geometric dimensions. Coupling with q-BICs: (**c**) Fano-like resonance under TE polarization and (**d**) EIT-like effect under TM polarization are observed.

As schematically illustrated in Figure 1a, an external optical pump can induce variations in the Ge conductivity, thereby modulating the overall electromagnetic properties of the metasurface. To break the in-plane symmetry, a portion of each cylinder is removed along the y-direction. The asymmetric Ge resonators are arranged with a lattice period of *p*, a radius of *R*, and a height of *h* (Figure 1b). The truncation boundary is parallel to the *x*-axis and separated from the cylinder center by a distance *d*. All electromagnetic simulations were performed using the Eigenfrequency and Frequency Domain study interfaces in the Wave Optics Module of COMSOL Multiphysics. The relevant simulation settings are summarized in Appendix A. In the considered spectral range of 1.1–1.3 THz, the relative permittivity of Ge and SiO_2_ are set to 16 and 3.4, respectively [19,28,30]. Since the conductivity of optically excited Ge materials can exceed 10^3^ S/m, a conductivity range of 0–200 S/m was selected to appropriately represent the photocarrier-generation process induced by external intensity-tunable optical pumping in the present study [28,29,30,31].

It is noteworthy that, upon breaking the structural symmetry, the symmetry-protected BICs are converted into q-BICs that can couple to external radiation channels under both TE- and TM-polarized excitation. Nevertheless, the two polarization configurations exhibit different resonance-formation mechanisms. As illustrated in Figure 1c, under TE-polarized excitation, the narrowband q-BIC resonance coherently interferes with a broadband background scattering channel, thereby producing a characteristic asymmetric Fano line shape in the transmission spectrum. In contrast, under TM-polarized excitation, the q-BIC mode couples coherently with a relatively bright mode resonance. The interference between the bright mode and a dark state gives rise to a high-Q EIT-like transmission window (Figure 1d). By carefully engineering the geometric parameters of the metasurface, the Fano transmission window under TE polarization and the EIT-like transmission window under TM polarization can be spectrally aligned at the same frequency, enabling co-frequency polarization-multiplexed resonant responses with distinct spectral line shapes. Furthermore, the tunable optical response of Ge within the investigated spectral range allows the two transparency windows to be further manipulated, enabling their selective or simultaneous activation and deactivation.

**Figure 2:**
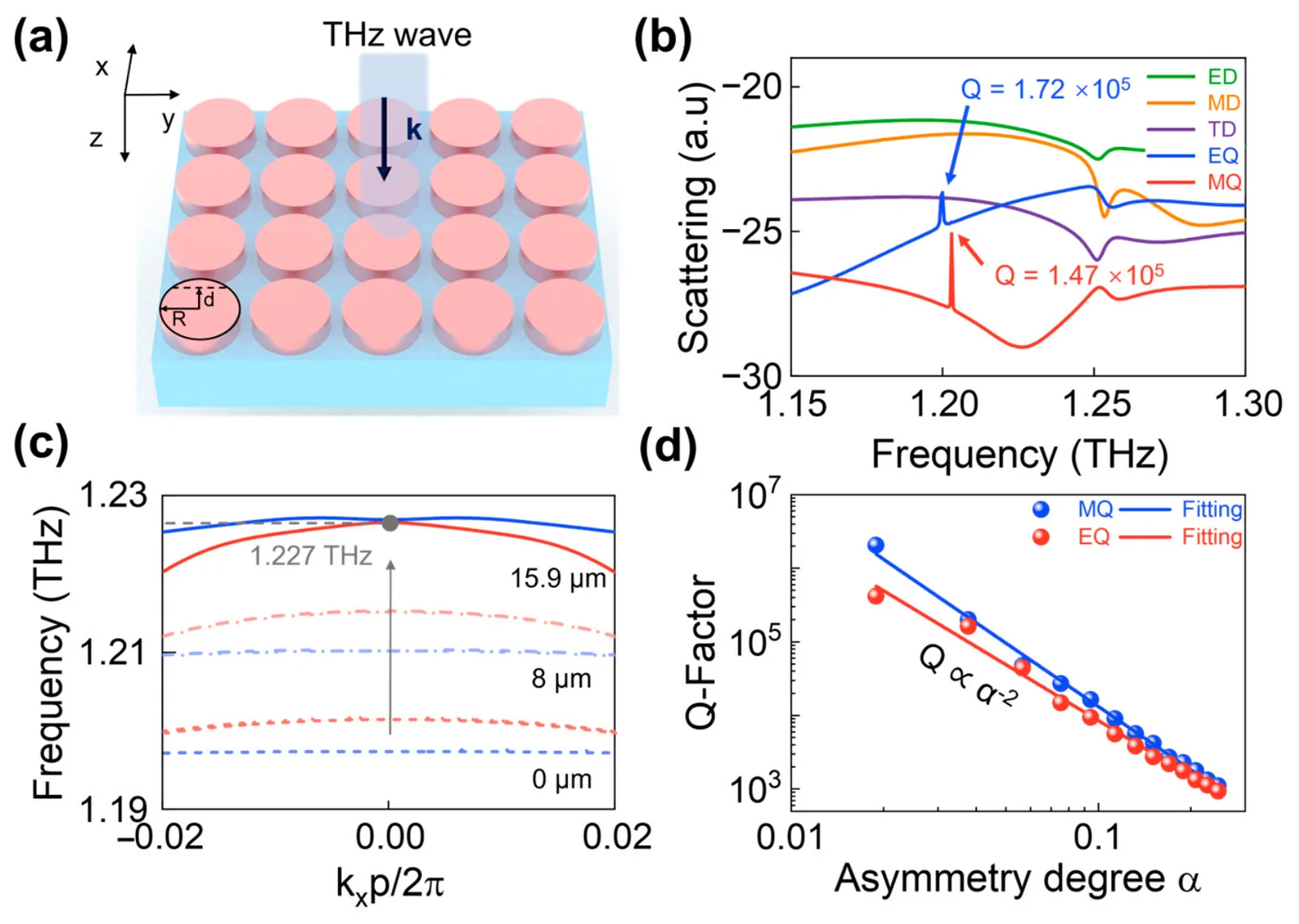
Mode characteristics of the metasurface unit cell. (**a**) Schematic of the symmetric unit cell. (**b**) Multipole decomposition of the Ge cylinder. (**c**) Mode evolution of the two modes with the introduction of asymmetry. (**d**) Q−factors of the two q-BICs as a function of asymmetry parameter.

To clarify the underlying mechanism of the presented metasurface, we begin by examining the symmetric configuration shown in Figure 2a. The simulations were performed using COMSOL Multiphysics. Figure 2b plots the unit cell diagram, and the geometric parameters are selected as *p* = 125 μm, *R* = 53 μm, *d* = 37.1 μm, and *h* = 31.8 μm. Multipole decomposition (the theoretical framework detailed in Appendix B) of the structure reveals two high-order resonant modes around 1.2 THz, which are predominantly characterized by an electric quadrupole (EQ) and a magnetic quadrupole (MQ), respectively. Both symmetry-protected modes exhibit radiative Q factors exceeding 1 × 10^5^ in the lossless simulation. Subsequently, keeping the height of the unit cell constant, we track the evolution of these two modes as the asymmetry factor increases, thereby leading to the band degeneracy of these two modes. As depicted in Figure 2c, an increasing value of R−d causes the two modes to gradually converge, ultimately becoming degenerate at 1.227 THz for 15.9 μm. Such behavior substantiates that the breaking of geometric symmetry effectively unlocks radiative channels, thereby transitioning the originally symmetry-protected modes into q-BICs. Furthermore, Figure 2d plots the Q factors against the asymmetry parameter α = (R−d) /R. The Q factors scale as $\alpha^{-2}$, which matches the characteristic dependence of symmetry-protected q-BICs.

## 3. Results and Discussion

### 3.1. Fano-like Resonance Under TE Polarization

**Figure 3:**
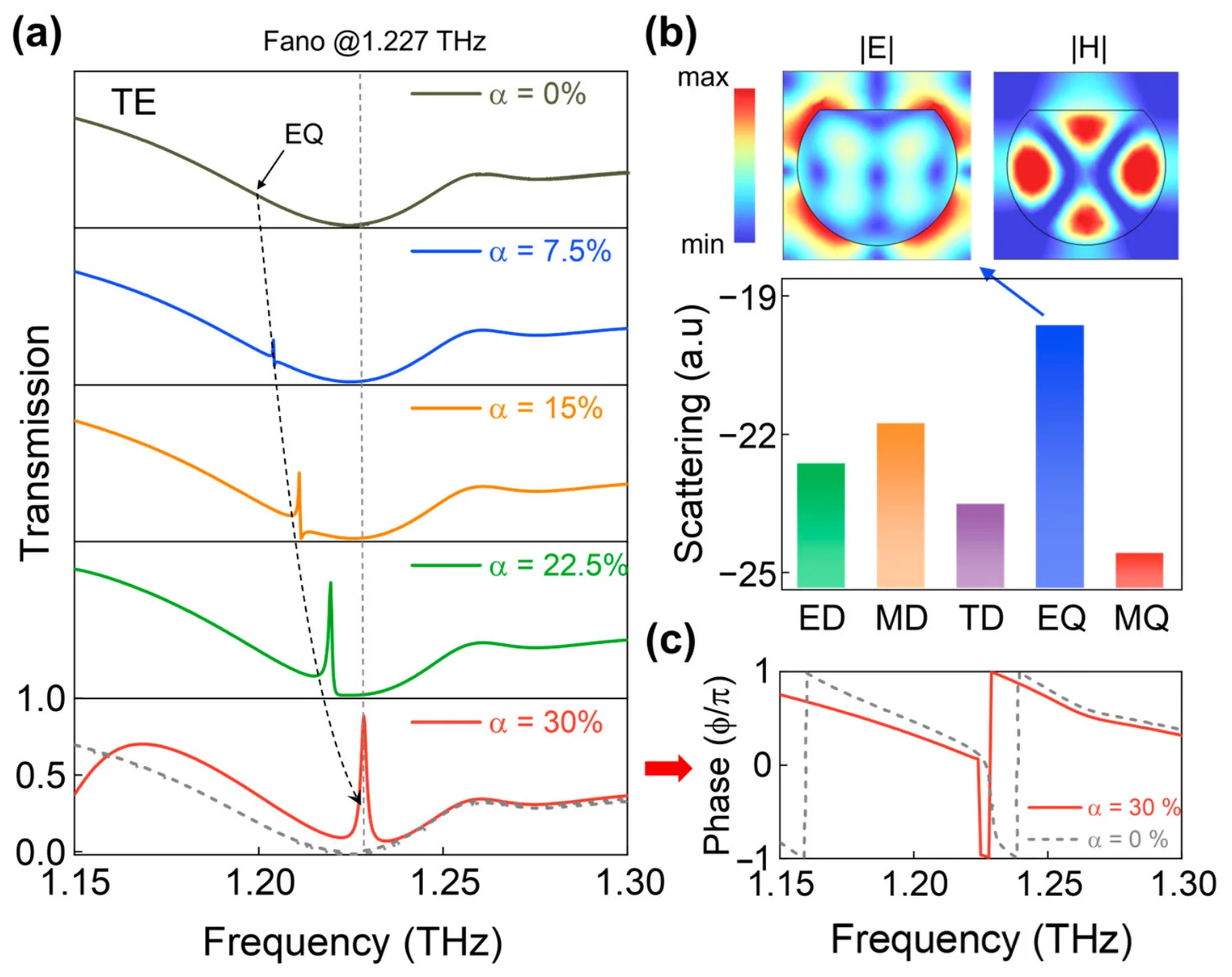
Fano-likeresonance characteristics of the proposed metasurface under TE polarization. (**a**) Transmission spectra variation against the asymmetry parameter. The gray dashed curve indicates the reference spectrum for the symmetric configuration. (**b**) Electric and magnetic field distributions at 1.227 THz (z = *h*/2) and the corresponding multipole decomposition. (**c**) Comparison of the transmitted phase for α of 0% and 30%.

Figure 3a presents the evolution of the transmission spectra under TE-polarized excitation over the frequency range of 1.15–1.30 THz as the α is increased. As the structural asymmetry gradually increases, the originally symmetry-protected EQ resonance progressively couples to the radiative continuum, leading to the emergence of a pronounced asymmetric Fano line shape in the transmission spectrum. The corresponding Fano-profile fitting is provided in Appendix C. Figure 3b shows the electric and magnetic field distributions within the meta-atom at 1.227 THz for α = 30%. The field distributions exhibit a pronounced antisymmetric character, indicating that the mode possesses an EQ radiation signature. This interpretation is further supported by the multipole decomposition, which identifies the EQ contribution as the dominant radiative component at the resonance. The corresponding phase response of the transmission coefficient is plotted in Figure 3c. A rapid and pronounced phase variation is observed near 1.227 THz, which is characteristic of coherent interference. It can be found that this strong phase dispersion coincides with the asymmetric spectral profile observed in the transmission spectrum.

### 3.2. EIT-like Resonance Under TM Polarization

**Figure 4:**
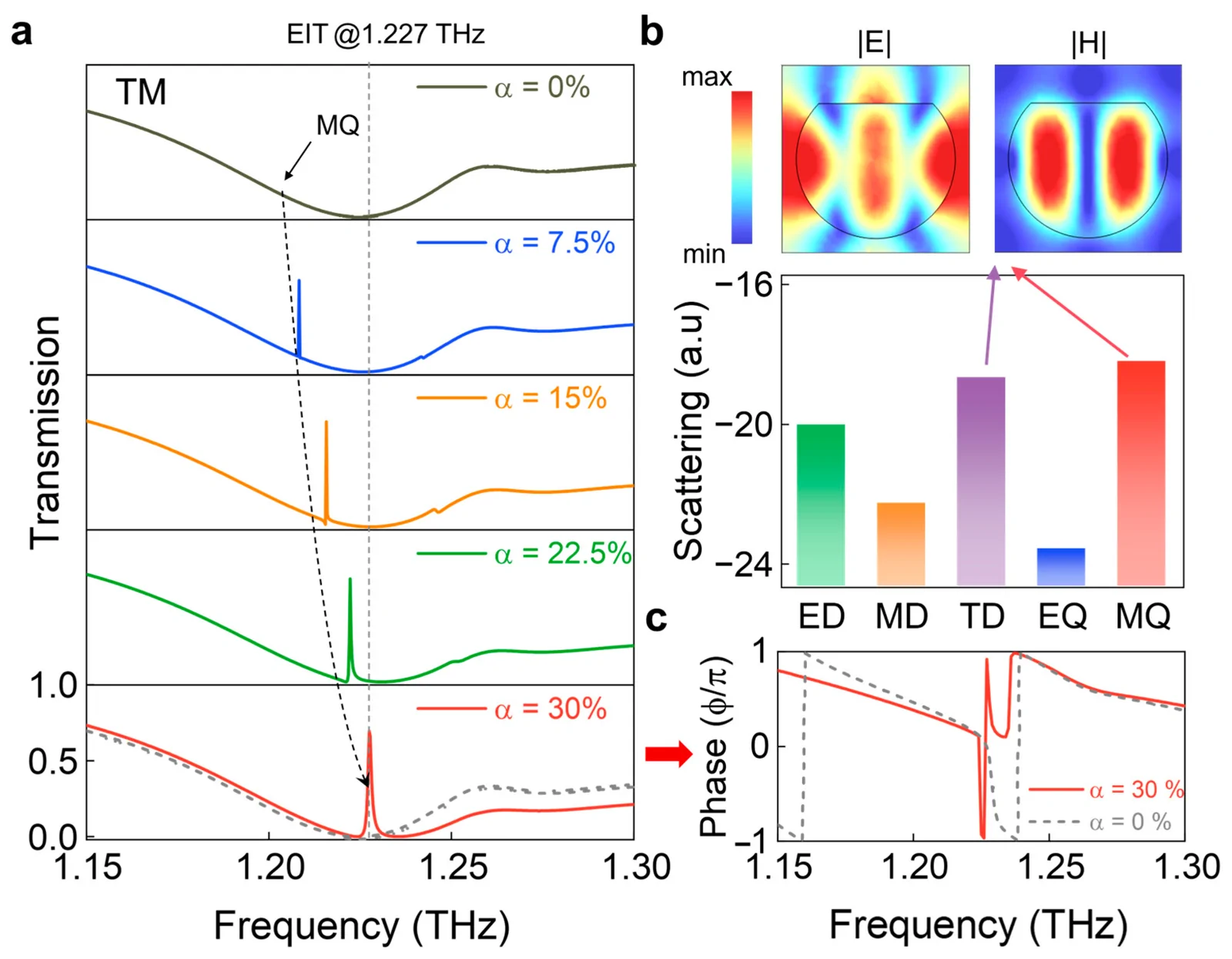
EIT-likeresonance characteristics of the proposed metasurface under TM polarization. (**a**) Transmission spectra variation against the asymmetry parameter. Similarly, the gray dashed curve indicates the reference spectrum for the symmetric configuration. (**b**) Electric and magnetic field distributions at 1.227 THz (z = *h*/2) and the corresponding multipole decomposition. (**c**) Comparison of the transmitted phase for α of 0% and 30%.

The transmission spectra under TM-polarized excitation are shown in Figure 4a. In contrast to the TE-polarized case, the progressive breaking of structural symmetry enables the originally symmetry-protected q-BIC mode to couple to the external radiation field, giving rise to finite radiative leakage from the otherwise dark mode. Similarly, Figure 4b shows the electric and magnetic field distributions. Compared with the relatively well-defined field pattern under TE polarization, the near-field distributions excited by TM polarization incidence are more intricate, suggesting the simultaneous involvement of multiple multipolar channels. To further identify the dominant contributions, we analyze the multipolar radiation intensities at 1.227 THz. Although the MQ remains the predominant contribution, the toroidal dipole (TD) and electric dipole (ED) also exhibit considerable intensities. These results indicate that the resonance arises from the collective response of several multipolar channels rather than from a single isolated multipole. Figure 4c depicts the frequency-dependent phase response. The transmission phase exhibits a steep dispersive variation within the narrow transmission window near 1.227 THz. Together with the interference feature observed in the transmission spectrum, this behavior is consistent with an EIT-like response and motivates the subsequent group-delay analysis.

### 3.3. Active Modulation and Group Delay Control

**Figure 5:**
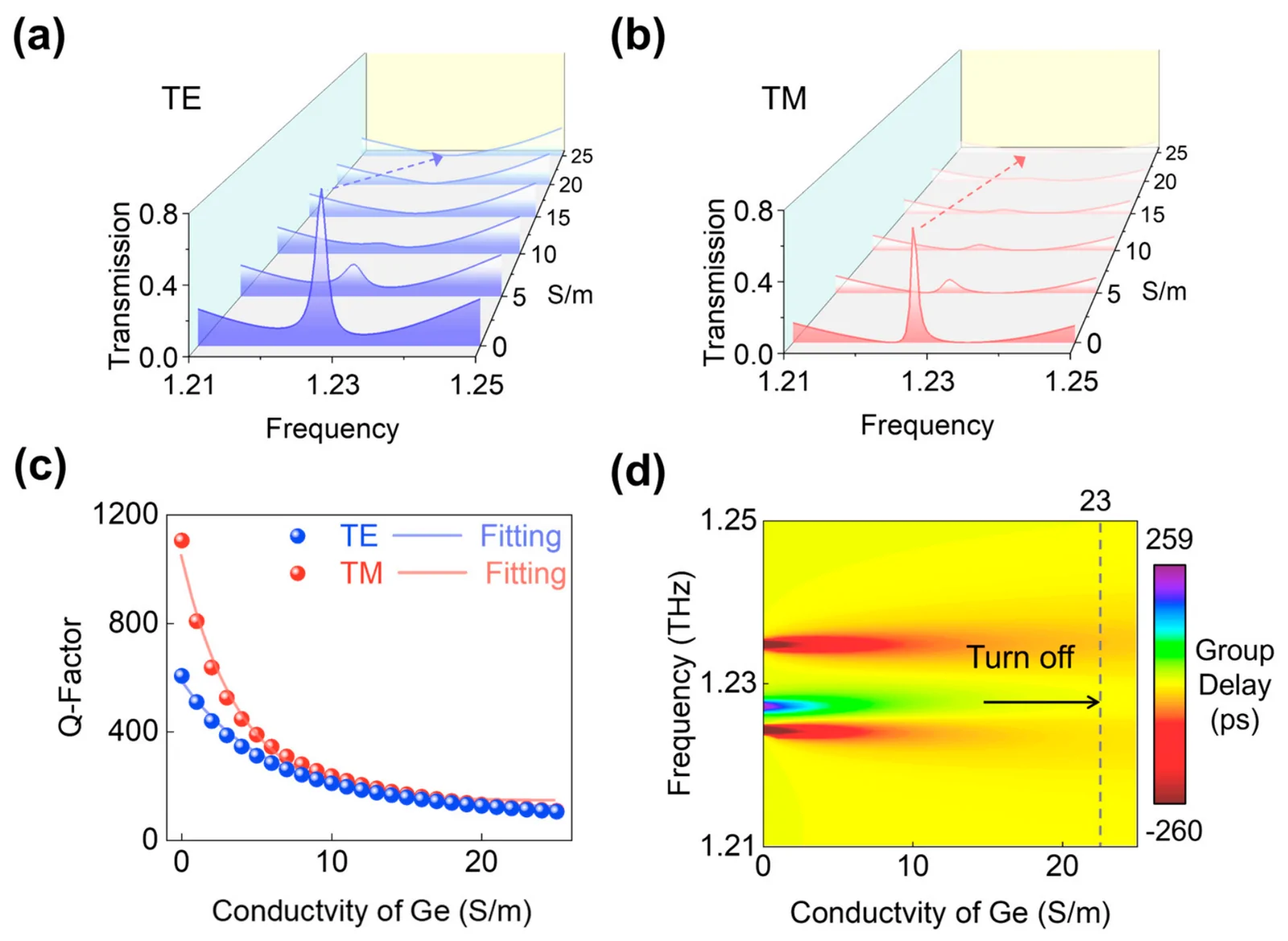
Modulation performance of the THz metasurface. Simulated transmission spectra with the Ge conductivity ranging from 0 to 25 S/m under (**a**) TE and (**b**) TM polarization. (**c**) The corresponding Q factors of the two resonances versus conductivity. (**d**) Colormap of the group delay as a function of frequency and conductivity under TM polarization.

Ge is a photoconductive semiconductor whose THz optical properties can be modified by external optical pumping. Under above-bandgap optical excitation, electron–hole pairs are generated in the Ge resonator, leading to an increase in the free-carrier concentration and, consequently, the electrical conductivity [5,28]. To further elucidate the active tunability of the proposed structure, we systematically investigate the evolution of the two resonances as a function of Ge conductivity while keeping all other structural and material parameters fixed. Under external optical pumping, the electrical conductivity of Ge increases with increasing pump intensity. This conductivity sweep represents the effect of increasing optical pump intensity on Ge. As the conductivity increases, both the Fano resonance (Figure 5a) and the EIT-like resonance (Figure 5b) weakened due to increased dissipative loss. At approximately 25 S/m, the resonant features became difficult to distinguish from the background. At this point, to quantify the modulation capability, the modulation depth (*MD*) is defined as *MD* = $\;(T_{on}-T_{off})/T_{on}$, where $T_{on}$ denotes the transmission amplitude without optical pumping, and $T_{off}$ represents the transmission amplitude when Ge is set to a given conductivity [5,28]. It can be observed that the maximum *MD*s under TE and TM polarizations reach 96.7% and 99.1% when the Ge conductivity attains 25 S/m, respectively. Additional spectral responses corresponding to different thicknesses of the optically excited Ge layer are presented in Appendix E. As shown in Figure 5c, the Q factors of the TE and TM modes decrease exponentially with increasing Ge conductivity. The corresponding exponential fits agree well with the simulated results, confirming that the high-Q resonances can be effectively regulated through Ge conductivity modulation.

Figure 5d presents the group-delay responses under different Ge conductivity values for TM polarization. The group delay is calculated using $\tau_{g}\;=\;d\phi /d\omega$, where ϕ denotes the phase and ω represents the angular frequency [23]. The maximum group delay of 259 ps occurs at 1.227 THz as the Ge is in an unexcited state. As the conductivity increases, the group-delay effect gradually diminishes to nearly zero as the resonance is switched off, and the suppression threshold is approximately 23 S/m. Table 1 summarizes recent studies and performance comparisons of metasurfaces featuring Fano or EIT resonances formed through BIC-related coupling. It can be found that the presented metasurface employs only a single resonator while supporting physically distinct q-BIC-derived mode responses under two orthogonal linear polarizations at the same operating frequency, without sacrificing a high Q factor. Moreover, both polarization channels can be almost completely switched on and off through conductivity modulation. This enables a tunable group delay in the TM channel, making the proposed metasurface particularly suitable for tunable THz optical devices.

To further clarify the distinct advantages of the two polarization channels, we investigated the potential biosensing application of the proposed metasurface in Appendix E. These results further demonstrate that the proposed metasurface is not polarization-independent in the conventional sense. Instead, it provides a co-frequency polarization-multiplexed platform in which two orthogonal polarization channels support different resonance line shapes and application functions at the same operating frequency. Such complementary responses may be useful for multifunctional THz biosensing, polarization-selective detection, and integrated photonic devices.

## 4. Conclusions

In summary, we have designed and numerically investigated a quasi-BIC-driven, co-frequency polarization-multiplexed, tunable all-dielectric THz metasurface. By introducing a single in-plane asymmetry parameter into a Ge resonator, distinct high-Q resonant responses are generated at the same operating frequency under two orthogonal linear polarizations: a Fano-type resonance under TE polarization and an EIT-like resonance under TM polarization. The two polarization-addressable channels exhibit complementary characteristics, with the TE channel providing a higher refractive-index sensitivity and the TM channel supporting a pronounced phase-dispersion response and a tunable group delay. Photoinduced conductivity modulation in Ge dynamically suppresses both resonances and enables control of their spectral amplitude, linewidth, and phase response. This single-resonator platform may provide a compact route toward co-frequency polarization-multiplexed THz devices for narrowband modulation, polarization-selective slow-light control, and dual-channel refractive-index sensing.

## Figures and Tables

**Table 1:** Recent comparative studies on the performance of metasurfaces employing BIC-related coupling to realize Fano or EIT resonances.

Metasurface	Resonator Number	Frequency /Wavelength	Polarization	Mode *	Q Factor	Max MD **	Group Delay	Ref
Al + GST	3	0.512 THz 0.703 THz	TE	Fano	/	>60%	/	[14]
Si	2	0.545 THz	TE	EIT	3802.61	/	1992.5 ps	[15]
Si + Graphene	1	2.505 THz 2.588 THz	TE	Fano	~10^5^	100% 36%	/	[19]
Si	1	1219 nm 1253 nm	TE	EIT	7674 3631	/	9.51 ps 5.69 ps	[22]
Si	1	~1370 nm	TE TM	EIT	/	/	/	[23]
Ge	1	1.227 THz	TE TM	Fano EIT	606 1105	~96.7% ~99.1%	/ 0~259 ps	This work

* The listed mode refers to the resonance generated or modified through coupling between a q-BIC and another resonant mode; ** ‘MD’ = Modulation Depth, ‘/’ indicates that the value was not reported.

**Figure A1:**
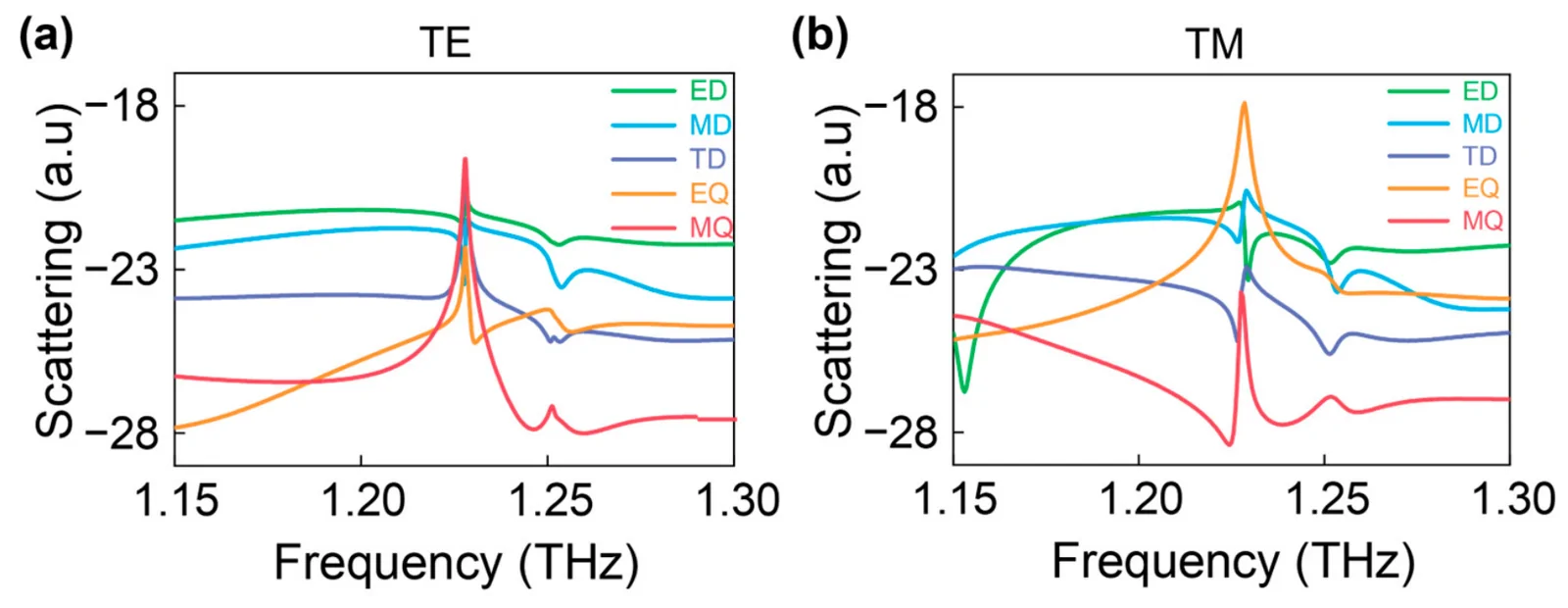
Scattered powers of (**a**) the Fano resonance under TE polarization, (**b**) the EIT resonance under TM polarization.

**Figure A2:**
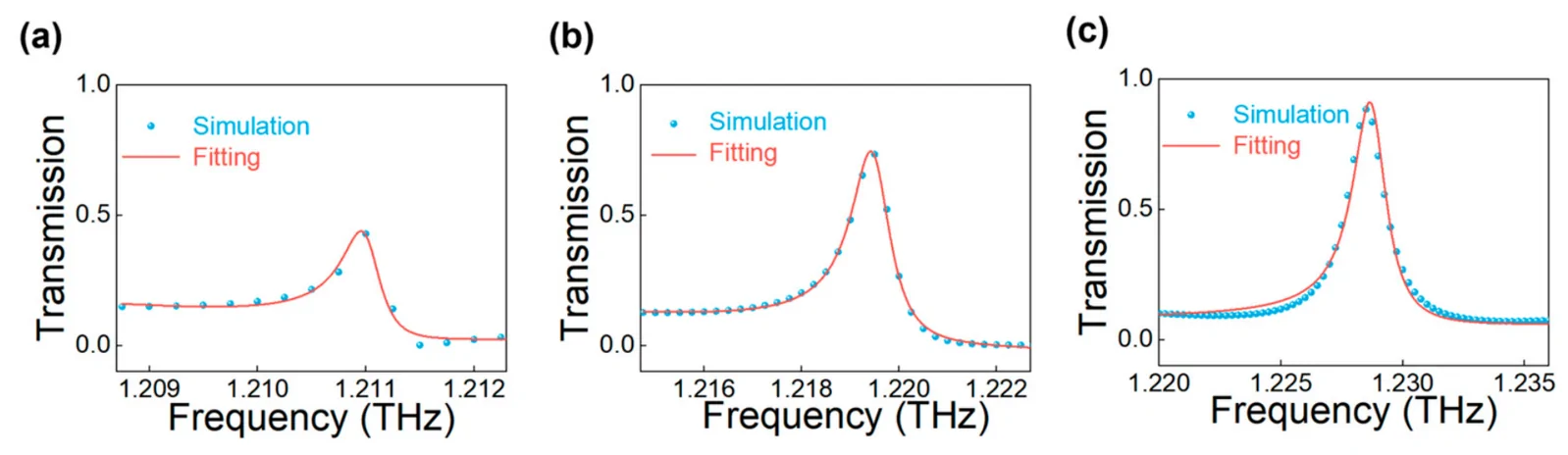
Fano fitting to the transmission spectrum with different asymmetries. (**a**) asymmetry of 8%; (**b**) asymmetry of 12%; (**c**) asymmetry of 16%.

**Figure A3:**
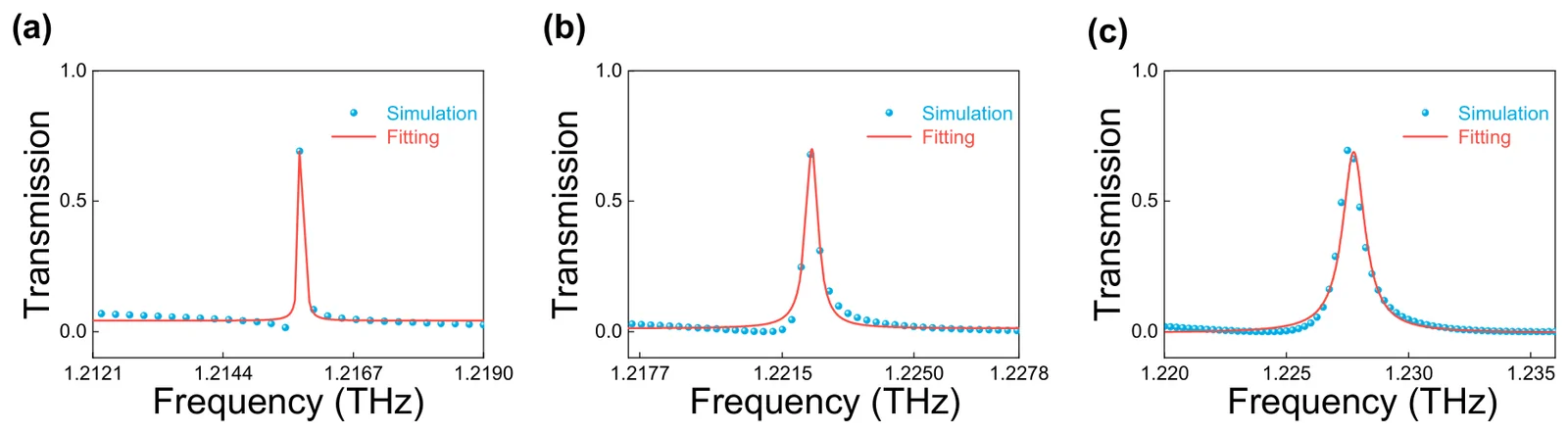
EIT fitting to the transmission spectrum with different asymmetries. (**a**) asymmetry of 15%; (**b**) asymmetry of 22.5%; (**c**) asymmetry of 30%.

**Figure A4:**
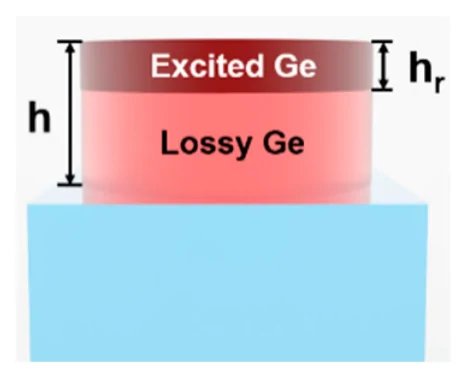
Schematic illustration of the depth-dependent photoexcitation model of the Ge metasurface element, consisting of an upper photoexcited Ge layer with thickness *h_r_* and a lower unexcited lossy Ge layer.

**Figure A5:**
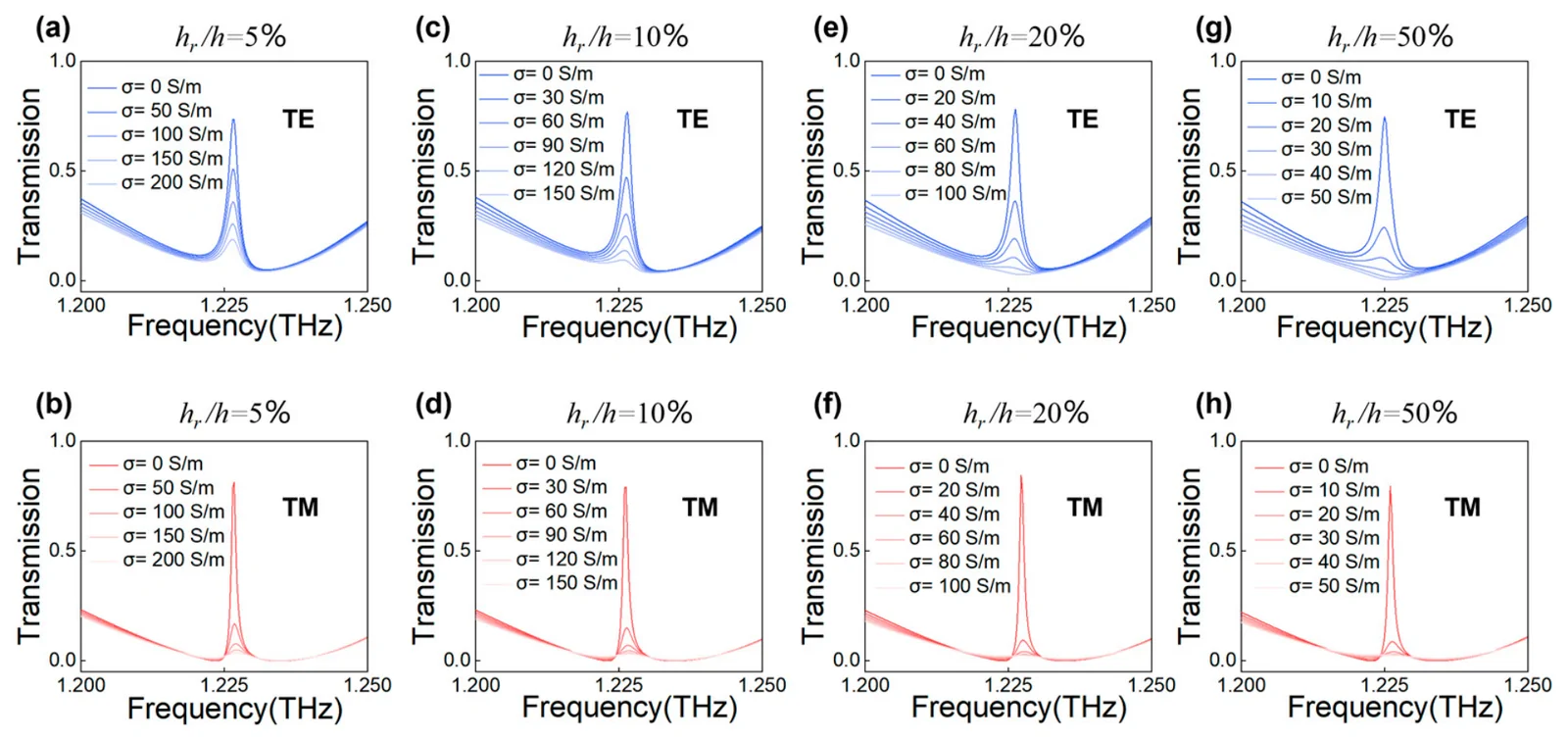
Simulated resonance responses under different photoexcited Ge thickness ratios and incident polarizations. (**a**,**c**,**e**,**g**) Fano resonance under TE polarization for excited thickness ratios *h*/*h_r_* = 5%, 10%, 20%, and 50%, respectively. (**b**,**d**,**f**,**h**) EIT resonance under TM polarization for the same ratios.

**Figure A6:**
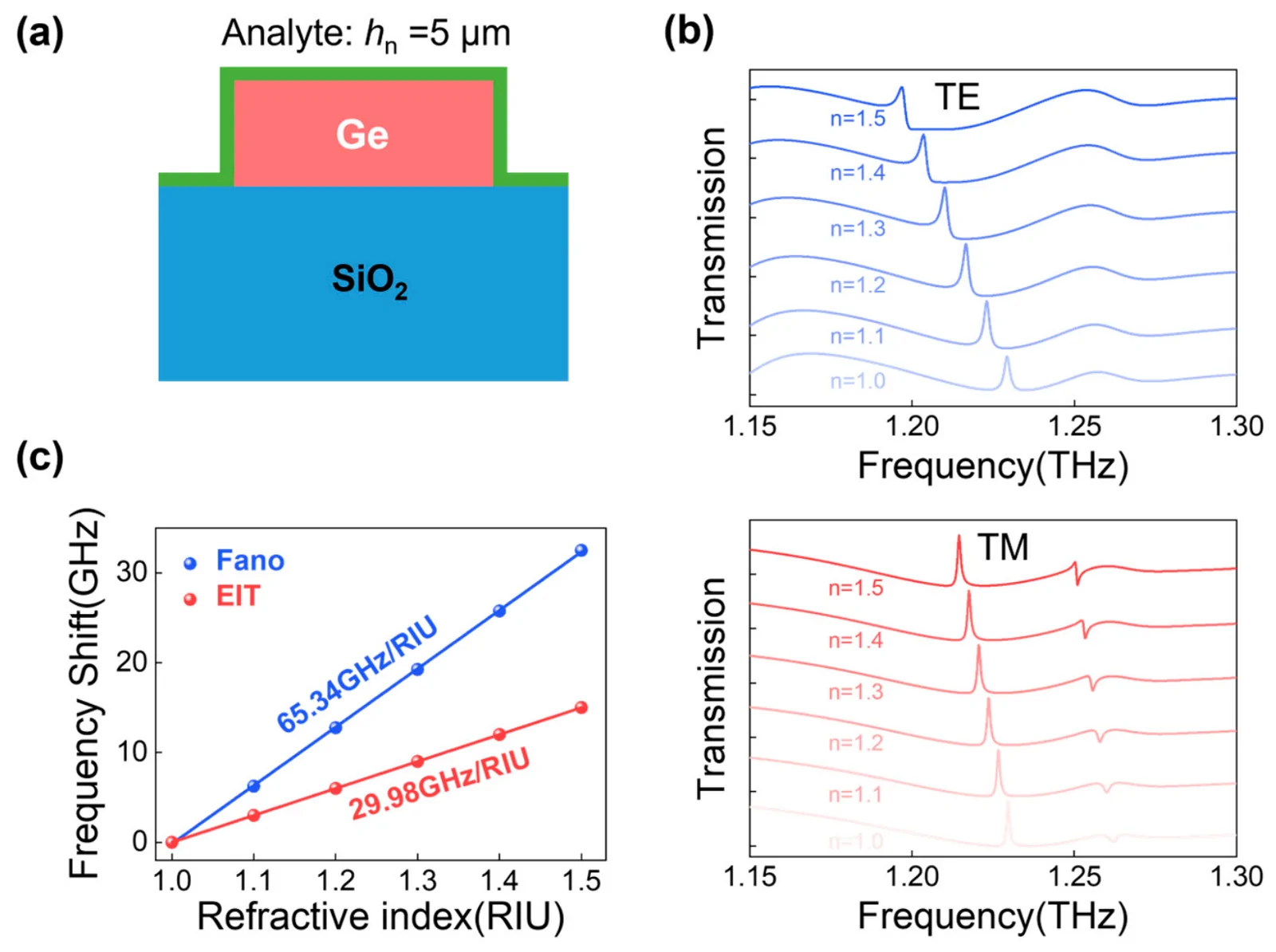
Biosensing performance of the proposed all-dielectric metasurface. (**a**) Schematic illustration of the analyte layer covering the metasurface. (**b**) Transmission spectra of the Fano and EIT resonance for different analyte refractive indices. (**c**) Linear fitting of the resonance frequency as a function of the analyte refractive index under two orthogonal polarizations. TE: y = −65.17 + 65.34x. TM: y = −29.97 + 29.98x.

**Figure A7:**
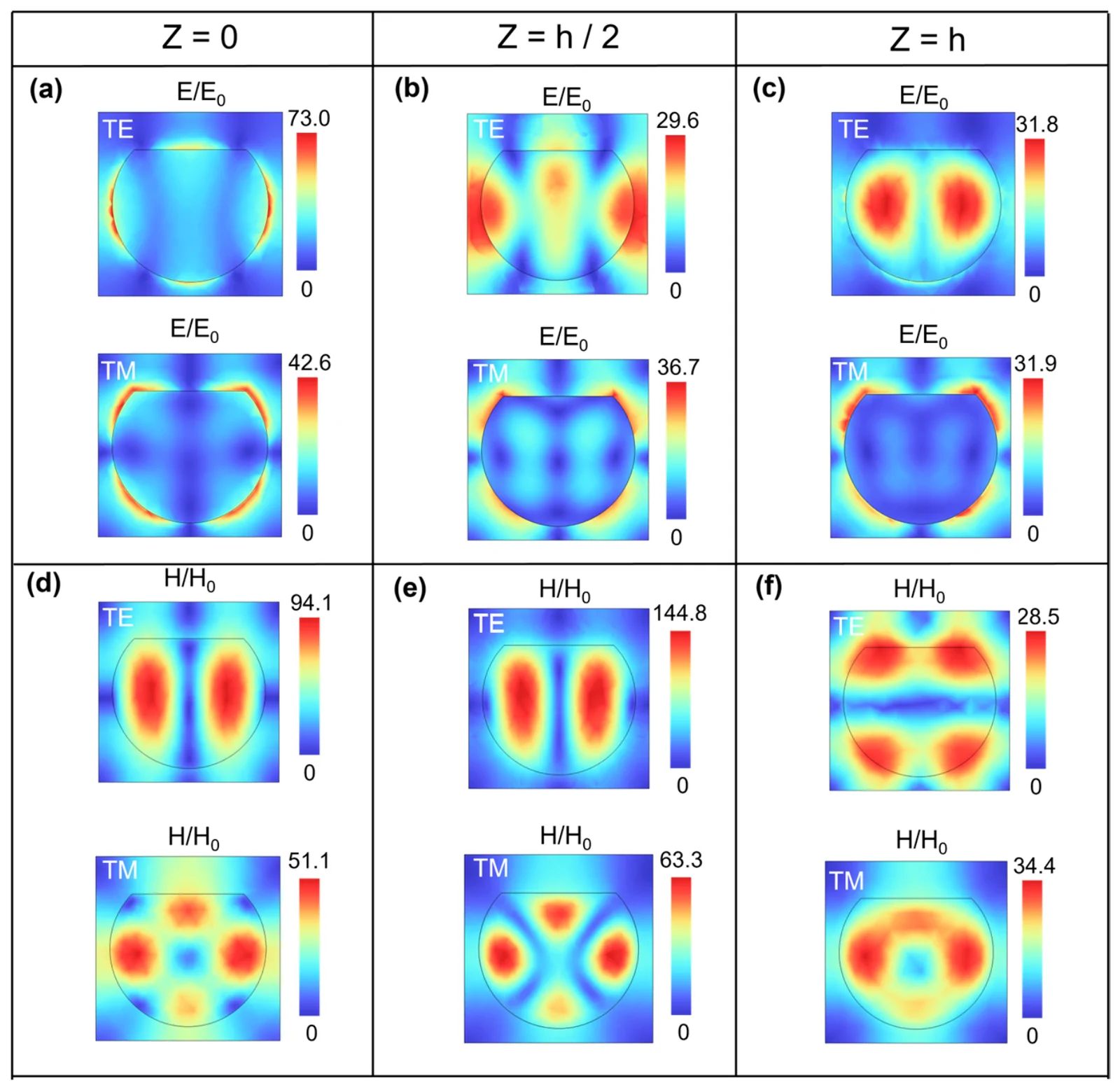
Simulated local electric- and magnetic-field enhancement distributions of the metasurface at 1.227 THz under TE and TM polarizations. The field distributions are shown in the planes *z* = 0 (**a**,**d**), *z* = *h*/2 (**b**,**e**), *z* = *h* (**c**,**f**), illustrating the spatial overlap between the enhanced near fields and the analyte layer.

## Data Availability

The data are available upon reasonable request from the corresponding author.

## Appendix A. Full Wave Simulation and Boundary Conditions

All electromagnetic simulations were performed using COMSOL Multiphysics 6.0. The Eigenfrequency and Frequency Domain functionalities of the Electromagnetic Waves, Frequency Domain (emw) interface are used to analyze the resonant modes and the driven electromagnetic response, respectively. The metasurface is modeled as a single unit cell, with Floquet-periodic boundary conditions applied along the two in-plane directions to represent an infinite periodic array.

For the frequency-domain calculations, the structure is excited at normal incidence through a Floquet port placed above the metasurface. The incident electric field is oriented along the polarization directions defined as TE and TM in the manuscript. To represent optical excitation, different conductivity values are assigned to the photoexcited Ge region, corresponding to different carrier-excitation levels. The resulting electromagnetic response is then calculated for each conductivity value.

## Appendix B. Multipole Decomposition Analysis

To analyze the mode composition of the Fano resonance under TE polarization and the EIT resonance under TM polarization, the Cartesian multipolar decomposition is carried out in this work. According to classical electromagnetic multipole expansion theory, the scattered field can be expressed as a superposition of multipole moments including electric dipole (ED), magnetic dipole (MD), toroidal dipole (TD), electric quadrupole (EQ), and magnetic quadrupole (MQ). The expressions for the corresponding multipole moments are given as follows:

(A1) $$P=\frac{1}{i\omega }\int Jd^{3}r$$

(A2) $$M=\frac{1}{2c}\int \left(r\times J\right)d^{3}r$$

(A3) $$T=\frac{1}{10c}\int \left[(r\cdot J)r-2r^{2}J\right]d^{3}r$$

(A4) $$Q_{\alpha \beta }=\frac{1}{2i\omega }\int \left[r_{\alpha }J_{\beta }+r_{\beta }J_{\alpha }-\frac{2}{3}(r\cdot J)\delta_{\alpha \beta }\right]d^{3}r$$

(A5) $$M_{\alpha \beta }=\frac{1}{3c}\int \left[{\left(r\times J\right)}_{\alpha }r_{\beta }+{\left(r\times J\right)}_{\beta }r_{\alpha }\right]d^{3}r$$

where *c* denotes the speed of light, ω denotes the frequency of light, α and β represent the *x*-, *y*- and *z*-directions of the coordinate axes [19,32].

The computing formulas of the far-field scattered power corresponding to each polar moment are as follows:

(A6) $$\mathrm{ED}=\frac{2\omega^{4}}{3c^{3}}|P{|}^{2}$$

(A7) $$\mathrm{MD}=\frac{2\omega^{4}}{3c^{3}}|M{|}^{2}$$

(A8) $$\mathrm{TD}=\frac{2\omega^{6}}{3c^{5}}|T{|}^{2}I_{Q}^{e}$$

(A9) $$\mathrm{EQ}=\frac{\omega^{6}}{5c^{5}}|Q_{\alpha \beta }{|}^{2}$$

(A10) $$\mathrm{MQ}=\frac{\omega^{6}}{40c^{5}}|M_{\alpha \beta }{|}^{2}$$

By comparing the variations in the scattering power of each multipole moment under TE and TM polarizations, the dominant modes of the Fano and EIT resonances under different polarization conditions can be identified, as shown in Figure A1, providing a theoretical basis for explaining the polarization-selective resonance mechanism in the designed structure.

## Appendix C. Lineshape Fitting

To quantitatively characterize the resonance lineshapes at different structural asymmetries, the transmission spectra of both the Fano and EIT resonances were fitted. Since the Fano lineshape model has been widely used to describe asymmetric resonances and has also been applied to the fitting of both Fano and EIT spectra, the same Fano lineshape equation was adopted here for both resonances [33,34]. The classical Fano fitting formula can be expressed as:

(A11) $$T=A_{0}+A\cdot \frac{{\left(q+\varepsilon \right)}^{2}}{1+\varepsilon^{2}},\varepsilon =\frac{\omega -\omega_{0}}{FWHM}$$

where ω denotes the frequency is the transmission coefficient; $\omega_{0}$ is the resonant frequency, and *FWHM* is the full width at half-maximum. The parameter *q* is the Fano asymmetry factor; *A* is the proportionality factor of the resonant amplitude, and *A*_0_ denotes the transmittance baseline [19,35]. The Fano parameters used in the fitting process are shown in Table A1.

**Table A1:** Fitting Fano parameters for transmission spectra with different asymmetries.

Asymmetry	*A*_0_	*A*	*q*	$\omega_{0}$	*FWHM*
8%	31.774–25.876 ω	0.03066	−3.44	1.2110	0.00021
12%	14.174–11.599 ω	0.01739	−6.34	1.2195	0.00049
16%	0.289–0.182 ω	0.00945	−9.43	1.2286	0.00083

Fano fitting was performed on three transmission spectra with different degrees of asymmetry as shown in Figure A2, specifically 8%, 12%, and 16%.

The EIT fitting was applied to the transmission spectra with asymmetry degrees of 15%, 22.5%, and 30%, as presented in Figure A3.

**Table A2:** Fitting EIT parameters for transmission spectra with different asymmetries.

Asymmetry	*A*_0_	*A*	*q*	$\omega_{0}$	*FWHM*
15%	1.42036–0.60878 ω	−0.64191	−0.03163	1.21579	0.000028
22.5%	0.77491–0.06670 ω	−0.68759	−0.00134	1.22229	0.00039
30%	0.70217–0.01420 ω	−0.69218	−0.00016	1.22775	0.00111

Through fitting the curves, a series of parameters of the resonant peak can be obtained, and these parameters provide an important basis for understanding the physical mechanisms of the metasurface.

## Appendix D. Influence of the Partially Excited Ge Thickness on Fano and EIT Resonance Tuning

Because Ge has an indirect bandgap of approximately 0.66 eV at room temperature, the photon energy of an 800 nm pump beam, approximately 1.55 eV, is sufficient to generate electron-hole pairs in Ge through optical excitation. However, owing to the finite penetration depth and absorption of the pump radiation in Ge, the 800 nm light may not completely penetrate the entire 31.8 μm thickness Ge layer. Consequently, the Ge metasurface element can be considered as a vertically stratified structure consisting of an upper photoexcited Ge layer and a lower unexcited, lossy Ge layer. This depth-dependent excitation model accounts for the incomplete penetration of the pump beam and its influence on the THz response, as illustrated in Figure A4.

Considering the unclear skin depth of the femtosecond pump beam in Ge, we simulated scenarios where only the top regions of the Ge layer were photoexcited with different excitation thickness ratios (*h*/*h_r_* = 5%, 10%, 20%, and 50%). These simulations were performed to evaluate the influence of localized carrier distributions on the modulation of the Fano resonance in the TE mode and the EIT resonance in the TM mode. The results are shown in Figure A5.

## Appendix E. Analysis of the Refractive-Index Sensing Applications

THz waves are highly sensitive to changes in the concentration and dielectric properties of biological materials, including cells [36]. To represent an analyte layer with a thickness comparable to the characteristic size of a cellular layer, we analyzed the sensitivity of the designed metasurface under two orthogonal polarizations, with a 5 μm-thick analyte layer completely covering the entire metasurface, as shown in Figure A6a. The analyte is modeled as a homogeneous, isotropic, and lossless dielectric layer, whose refractive index is varied from 1 to 1.5. As the refractive index of the analyte increases, the corresponding changes in the transmission spectra are plotted in Figure A6b. It can be observed that as the refractive index of the analyte increases, the resonance peaks under both TE and TM polarizations exhibit a red shift. Accordingly, the sensitivity can be obtained from the slope of the resonance frequency as a function of the refractive index:

$$S_{f}=\frac{\Delta f}{\Delta n}$$

where Δn and Δf represent the change in analyte refractive index and the corresponding shift in resonance peak frequency, respectively. Based on linear fitting using the regression-line equation, the sensitivities under the two polarizations are approximately 65.34 and 29.98 GHz/RIU, respectively, which are plotted in Figure A6c. Interestingly, despite its lower Q factor, the Fano resonance under TE polarization exhibits a higher frequency sensitivity than the EIT-like resonance under TM polarization.

To further explain this phenomenon, the field enhancement of the metasurface under TE- and TM-polarized electromagnetic wave incidence at the resonant frequency of 1.227 THz is analyzed. At this point, the enhancement factor of the electric or magnetic field is defined as $\left|E/E_{0}\right|$ and $\left|H/H_{0}\right|$, respectively. E and H represent the electric and magnetic field distributions with a metasurface, while $E_{0}$ and $H_{0}$ denote the case without the meta-atom. Figure A7 plots the enhancement factor map, with the observation plane located at z = 0, *h*/2 and *h*. Under both TE and TM polarizations, the local electric and magnetic fields exhibit enhancement factors on the order of tens. These results further confirm the significant localized electromagnetic-field enhancement effect of the designed metasurface, which can strengthen the interaction between an analyte layer placed on the metasurface surface and the optical field, thereby improving the biosensing performance. Furthermore, it can also be observed that the TE mode shows stronger electric-field overlap with the analyte than the TM mode in the present structure. These results indicate that the sensitivity of the metasurface depends not only on the Q factor but also on the spatial overlap between the enhanced electric field and the analyte.
